# Relationships of the Trace Elements Zinc and Magnesium With Diabetic Nephropathy-Associated Renal Functional Damage in Patients With Type 2 Diabetes Mellitus

**DOI:** 10.3389/fmed.2021.626909

**Published:** 2021-03-30

**Authors:** Jianan Feng, Heyuan Wang, Zhe Jing, Yue Wang, Wanning Wang, Yanfang Jiang, Weixia Sun

**Affiliations:** ^1^Department of Nephrology, The First Hospital of Jilin University, Changchun, China; ^2^Department of Blood Purification, Beijing Chao-Yang Hospital, Capital Medical University, Beijing, China; ^3^Department of Endocrinology and Metabolism, The First Hospital of Jilin University, Changchun, China; ^4^Department of Laboratory Medicine, The First Hospital of Jilin University, Changchun, China; ^5^Genetic Diagnosis Center, The First Hospital of Jilin University, Changchun, China

**Keywords:** type 2 diabetes mellitus, diabetic nephropathy, trace element, zinc, magnesium, pro-inflammatory cytokine

## Abstract

Zinc (Zn) and magnesium (Mg) are essential trace elements in humans. Their deficiency may be associated with inflammation and oxidative stress (OS) in patients with diabetic nephropathy (DN), but the mechanisms involved have not been fully characterized. We aimed to investigate the relationships between circulating concentrations of Zn and Mg and pro-inflammatory factors with DN-associated renal functional damage in patients with type 2 diabetes mellitus (T2DM). To this end, we studied 20 healthy people, 24 patients with T2DM, and 59 patients with T2DM and T2DN. Serum and urine Zn and Mg concentrations were measured using the 2-(5-nitro-2-pyridylazo)-5-(N-propyl-N-sulfopropylamine) phenol (nitro-PAPS) chromogenic method and the xylidyl blue method, respectively, and the circulating concentrations of pro-inflammatory cytokines [interleukin (IL)-1β, IL-6, IL-8, IL-10, IL-12, and tumor necrosis factor-α (TNF-α)] were measured using flow cytometry. The serum concentrations of Zn and Mg were significantly lower in patients with T2DM and DN than in healthy controls. Serum Zn, urine Zn, and urine Mg concentrations decreased, while those of IL-6 and IL-8 increased with the progression of DN-associated renal functional damage. Furthermore, the serum and urine Zn concentrations negatively correlated with the serum IL-6 and IL-8 concentrations. Notably, the serum Zn concentration was found to independently protect against DN in patients with T2DM. Hypozincemia may be associated with the T2DN-associated renal functional damage because it exacerbates inflammation.

## Introduction

Diabetes mellitus (DM) is a well-established public health problem because of its high prevalence and morbidity (1). Diabetic nephropathy (DN) is one of the most important microvascular complications of DM. The principal pathological features of DN are glomerular sclerosis and interstitial fibrosis (2), and the mechanism is thought to involve the induction of oxidative stress (OS) (3), the activation of inflammatory and fibrosis signaling pathways (4), the accumulation of advanced glycation end-products (5), abnormal renal hemodynamics, and abnormal activation of the renin-angiotensin-aldosterone system, secondary to chronic hyperglycemia (6). In particular, OS and inflammation are thought to play important roles in the pathogenesis of DN (7, 8).

The trace elements zinc (Zn) and magnesium (Mg) play important roles in the function of various enzymes and transcription factors (9, 10). Zn has anti-inflammatory effects and is involved in the induction of antioxidant proteins (11). Many previous studies have shown Zn deficiency in patients with type 2 diabetes mellitus (T2DM) and DN, and in animal models of nephropathy (12, 13). Specifically, Li et al. (14) have shown that in diabetes, Zn promotes the dissociation of nuclear factor-erythroid 2-related factor 2 from Kelch-like ECH-associated protein-1 and its translocation to the nucleus, where it promotes the expression of downstream antioxidant genes. In addition, Barman et al. (13) showed that Zn administration inhibits activation of the nuclear factor kappa-B (NF-κB) signaling pathway, thereby reducing oxidative damage and the inflammatory response in the kidneys of rats with streptozotocin-induced diabetes.

Mg is not only involved in many important metabolic processes, but also has antioxidant, anti-inflammatory, and anti-apoptotic effects (15). Mg deficiency leads to impaired glucose tolerance, insulin resistance, abnormal lipid metabolism, and OS (10). Previous studies have shown that the prevalence of hypomagnesemia in patients with DN is significantly higher than that in those without (16). In addition, Mg deficiency leads to the opening of L-type calcium (Ca^2+^) channels in macrophage, which increases the intracellular Ca^2+^ concentration and promotes the release of pro-inflammatory cytokines. Conversely, supplementation of Mg reduces the production of pro-inflammatory cytokines (17).

In the present study, we aimed to determine (1) the predictors of DN in patients with T2DM; (2) the relationships between Zn and Mg concentrations and DN-associated renal functional damage in patients with T2DM; and (3) the relationships between Zn and Mg concentrations and pro-inflammatory cytokines in patients with T2DM.

## Materials and Methods

### Participants

Clinical data was collected for patients with T2DM and DN who were hospitalized in the nephrology and endocrinology department of the First Hospital of Jilin University between September 2017 and January 2019. At the same time, we recruited a group of healthy individuals who underwent check-ups at a physical examination center.

The inclusion criteria for the study were (1) conformation with the 1999 World Health Organization diagnostic criteria for T2DM and/or the 2007 National Kidney Foundation's Kidney Disease Outcomes Quality Initiative guidelines for the diagnosis of DN; (2) patients who were not undergoing dialysis; and (3) a complete set of clinical data. The exclusion criteria were (1) presence of a malignant tumor; (2) presence of severe cardiac insufficiency; (3) presence of non-diabetic nephropathy; (4) recent infection, fever, or immune-mediated disease; and (5) lack of compliance with the protocol; (6) use of dietary Zn and/or Mg supplements in the past 3 months.

### Diagnostic Criteria and Grouping

T2DM was defined using the World Health Organization 1999 criteria as follows: (1) presence of typical symptoms, with a random plasma glucose of ≥11.1 mmol/L, (2) fasting plasma glucose (FPG) ≥ 7.0 mmol/L, or (3) 2-h glucose concentration during an oral glucose tolerance test of ≥11.1 mmol/L. Individuals without typical symptoms had their plasma glucose measured on a second day. Individuals fulfilling one of the above criteria were diagnosed with diabetes. DN was diagnosed using the 2007 KDOQI guidelines as follows: (1) macroalbuminuria (albumin/creatinine ratio (ACR) >300 mg/g), (2) presence of diabetic retinopathy and microalbuminuria, or (3) duration of T2DM > 10 years and microalbuminuria.

The participants were allocated to the following groups according to the presence of T2DM and/or DN: (1) T2DM + DN group (*n* = 59), with ACR ≥30 mg/g; (2) T2DM group, with ACR <30 mg/g (*n* = 24); and (3) healthy control group (*n* = 20). Participants in the control group were normal on physical examination and did not have a family history of diabetes, cardiovascular disease, or serious liver or kidney disease.

According to the ACR and estimated glomerular filtration rate (eGFR), the participants with DN were further allocated to three subgroups as follows: (1) microalbuminuria group, with 30 ≤ ACR <300 mg/g (*n* = 18); (2) macroalbuminuria group, with ACR ≥ 300 mg/g (*n* = 24); and (3) renal failure group, in which the participants had an eGFR <15 ml min^−1^.1.73 m^−2^ but were not undergoing dialysis (*n* = 18).

### Clinical Data and Biochemical Parameters

Clinical data were collected from medical records and comprised sex, age, height, body mass, history of smoking, and history of hypertension.

Glycated hemoglobin (HbA1c) was measured using high-performance liquid chromatography (Variant II; Bio-Rad, CA, USA). FPG, blood urea nitrogen (BUN), serum creatinine (SCr), serum uric acid (UA), total cholesterol (TC), and triglyceride (TG) concentrations were measured using enzymic methods (7600-210 automatic biochemical analyzer; Hitachi, Tokyo, Japan). Twenty-four-hour urine albumin loss was measured using a turbidimetric immunoassay (BN II; Siemens, Marburg Germany). The pyrogallol red and amino acid oxidase methods were used to determine urinary albumin and creatinine concentrations, respectively, which were used to calculate the ACR.

### eGFR Calculation

eGFR was calculated using the Modification of Diet in Renal Disease formula as follows: eGFR (ml min^−1^ 1.73 m^−2^) = 186 × SCr (mg/dl)^−1.154^ × age (years)^−0.203^ ( ×0.742 for women).

### Measurement of Trace Element Concentrations

The concentrations of Zn and Mg were measured on a Hitachi 7600-210 automatic biochemical analyzer (Hitachi, Tokyo, Japan) using the PAPS chromogenic method (BEBS, Beijing, China) and the Xylidyl blue method (Leadman, Beijing, China), respectively.

### Measurement of Pro-Inflammatory Cytokine Concentrations

Plasma levels of IL-1β, IL-6, IL-8, IL-10, IL-12, and TNF-α were measured using the Cytometric Bead Array Human Inflammation kit (CBA; BD Biosciences, NJ, USA). Plasma was stored at −80°C until use. Six human inflammatory cytokine standard curves with concentrations standard ranging from 20 to 5,000 pg/ml were obtained using a set of standard solutions. The capture beads were added to the assay tubes and vortexed. Next, 50 μL of each plasma sample was added to the sample tubes and 50 μL of each human standard dilution was added to the control tubes. Then, 50 μL of PE detection reagent was added to all assay tubes and the tubes were incubated for 3 h. The results were acquired and the data were analyzed using Cell Quest software and FCAP Array software. C-reactive protein (CRP) concentration was measured using a turbidimetric immunoassay method on an AU5800 (Beckman Coulter).

### Statistical Methods

SPSS 22.0 (IBM, Inc., Armonk, NY, USA) was used to analyze the data. Normally distributed data are expressed as mean ± standard deviation (SD). One-way analysis of variance (ANOVA) was used to compare normally distributed data between multiple groups. Non-normally distributed data are expressed as median (range). The Mann–Whitney *U*-test was used to compare non-normally distributed data between two groups, and the Kruskal–Wallis test was used to compare such data between multiple groups. The chi-square test was used to compare ratios among groups. A bivariate regression model was used to identify risk factors for DN. The relationships of Zn, Mg, and cytokine concentrations with renal function were analyzed using Pearson's correlation for normally distributed data and Spearman's correlation for non-normally distributed data. *P* < 0.05 was regarded as statistically significant.

## Results

### Demographic and Biochemical Parameters of the Studied Population With and Without T2DM and T2DN

Of the 103 participants, 59 had T2DN, 24 had T2DM alone, and there were 20 controls. The T2DN group contained 39 men (66.1%) and had a mean age of 56.4 ± 7.3 years; the T2DM group contained 17 men (70.8%) and had a mean age of 52.7 ± 10.1 years; and the healthy control group contained 10 men (50%) and had a mean age of 53.0 ± 5.7 years. Table 1 shows that there were no significant differences between the T2DM, T2DM + T2DN, and control groups with respect to sex ratio, age, smoking history, serum TC, 24 h urine Mg loss, serum IL-12 concentration, or serum TNF-α concentration. The T2DM + T2DN and T2DM groups had a higher body mass index (BMI) and higher FPG, HbA1c, 24 h urine Zn, and serum CRP, IL-6, and IL-10 concentrations, as well as lower serum Mg and Zn concentrations than the control group (*P* < 0.05). The T2DM + T2DN group contained more participants with hypertension; had higher BUN, SCr, UA, TC, IL-6, and IL-8 concentrations; and had lower serum Zn and eGFR than the T2DM and healthy control groups (*P* < 0.05). Finally, the T2DM + T2DN group had lower 24 h urine Zn loss, and serum IL-1β and IL-10 concentrations, than the T2DM group (*P* < 0.05).

**Table 1:** Demographic and biochemical parameters of the studied population with and without T2DM and T2DN.

**Item**	**Control group** ** (*n* = 20)**	**T2DM group** ** (*n* = 24)**	**T2DM+T2DN group** ** (*n* = 59)**	***P***
Male, *n* (%)	10 (50.0%)	17 (70.8%)	39 (66.1%)	0.316
Age (years)	53.00 ± 5.69	52.67 ± 10.08	56.42 ± 7.32	0.064
Hypertension, *n* (%)	4 (20.0%)	11 (45.8%)	51 (86.4%)^*^^&^	<0.001
Smoking, *n* (%)	5 (25.0%)	9 (39.1%)	22 (38.9%)	0.518
BMI (kg/m^2^)	23.25 ± 3.59	25.64 ± 2.87^*^	26.68 ± 3.29^*^	<0.001
BUN (mmol/L)	4.92 (4.58–5.49)	5.75 (4.55–7.35)	9.12 (7.35–16.62)^*^^&^	<0.001
SCr (μmol/L)	60.5 (51.25–77.3)	63.80 (56.00–72.40)	162.70 (76.40–411.80)^*^^&^	<0.001
UA (mmol/L)	319.00 ± 84.52	327.52 ± 71.04	407.31 ± 75.21^*^^&^	<0.001
FPG (mmol/L)	4.69 (4.35–5.25)	8.30 (6.11–9.59)^*^	7.20 (5.19–10.54)^*^	<0.001
HbA1c (%)	5.44 ± 0.43	8.28 ± 1.75^*^	7.54 ± 1.97^*^	<0.001
TC (mmol/L)	5.21 (4.57–5.92)	4.66 (3.91–5.43)	5.26 (4.44–6.55)	0.108
TG (mmol/L)	1.41 (1.00–1.86)	2.02 (1.07–2.87)	2.68 (1.57–4.13)^*^^&^	<0.001
Serum Mg (mmol/L)	0.96 ± 0.05	0.89 ± 0.08^*^	0.85 ± 0.09^*^	<0.001
Serum Zn (mmol/L)	13.70 ± 1.67	10.32 ± 2.89^*^	8.20 ± 2.70^*^^&^	<0.001
24 h urine Mg (mmol/L)	3.02 ± 0.19	3.64 ± 1.40	3.02 ± 1.35	0.107
24 h urine Zn (mmol/L)	4.92 ± 1.22	10.67 ± 4.10^*^	7.55 ± 4.93^*^^&^	<0.001
IL-1β (pg/mL)	1.86 (1.16–3.35)	2.07 (1.30–2.88)	1.11 (0.74–2.55)^&^	0.038
IL-6 (pg/mL)	1.52 (1.27–2.07)	3.43 (2.49–4.03)^#x0002A;^	5.77 (3.23–9.64)^*^^&^	<0.001
IL-8 (pg/mL)	5.50 ± 1.19	5.10 ± 1.41	7.27 ± 2.58^*^^&^	<0.001
IL-10 (pg/mL)	1.00 ± 0.61	2.11 ± 0.79^*^	1.57 ± 0.70^*^^&^	<0.001
IL-12 (pg/mL)	0.39 (0.00–0.97)	0.08 (0.00–0.83)	0.28 (0.00–0.60)	0.525
CRP (mg/L)	3.02 (3.02–3.02)	3.23 (3.13–3.23)^*^	3.23 (3.13–4.08)^*^	<0.001
TNF-α (pg/mL)	1.31 ± 1.01	1.75 ± 1.15	1.56 ± 0.77	0.056
eGFR (ml min^−1^ 1.73 m^−2^)	117.75 (95.20–127.55)	116.78 (92.51–138.72)	41.13 (13.62–81.58)^*^^&^	<0.001

* vs. the control group P < 0.05

& *vs. the T2DM group P < 0.05. P < 0.05 means a statistically significant difference. BMI, body mass index; BUN, blood urea nitrogen; SCr, serum creatinine; UA, uric acid; FPG, fasting plasma glucose; HbA1c, hemoglobin A1c; TC, total cholesterol; TG, triglyceride; Zn, zinc; Mg, magnesium; IL, interleukin; CRP, C-reactive protein; TNF-α, tumor necrosis factor-α; eGFR, estimated glomerular filtration rate; T2DM, type 2 diabetes mellitus; DN, diabetic nephropathy*.

### Predictors of DN

#### Predictors for DN Identified by Logistic Regression Analysis, Adjusted for a History of Hypertension

Table 2 shows the results of logistic regression analysis to identify predictors for T2DN. Single-factor logistic regression analysis showed that BMI, a history of hypertension, FPG, HbA1c, and the serum TC, IL-6, and IL-10 concentrations were risk factors; whereas, the serum Zn and Mg concentrations and eGFR were protective. Using these findings, we conducted multiple factor analysis, which showed that FPG and IL-10 are risk factors for T2DN, and that Zn is protective. These three factors were considered independent predictors of T2DN using a model adjusted for a history of hypertension.

**Table 2:** Predictors of DN identified by logistic regression analysis, adjusted for a history of hypertension.

	**Model 1**			**Model 2**		
	**OR**	**95%CI**	***P***	**OR**	**95%CI**	***P***
FPG (mmol/L)	3.018	1.106–8.239	0.031	6.291	1.476–17.017	0.013
Serum Zn (mmol/L)	0.295	0.111–0.784	0.014	0.241	0.074~0.086	0.018
IL-10 (pg/mL)	12.445	1.552–19.672	0.032	6.881	1.498–27.017	0.013

*P < 0.05 indicates a statistically significant difference. FPG, fasting plasma glucose; Zn, zinc; IL-10, interleukin-10*.

*Model 1: Unadjusted*.

*Model 2: Adjusted for a history of hypertension*.

#### Sensitivity and Specificity of Predictors of DN by Receiver Operating Characteristic Curve Analysis

**Figure 1:**
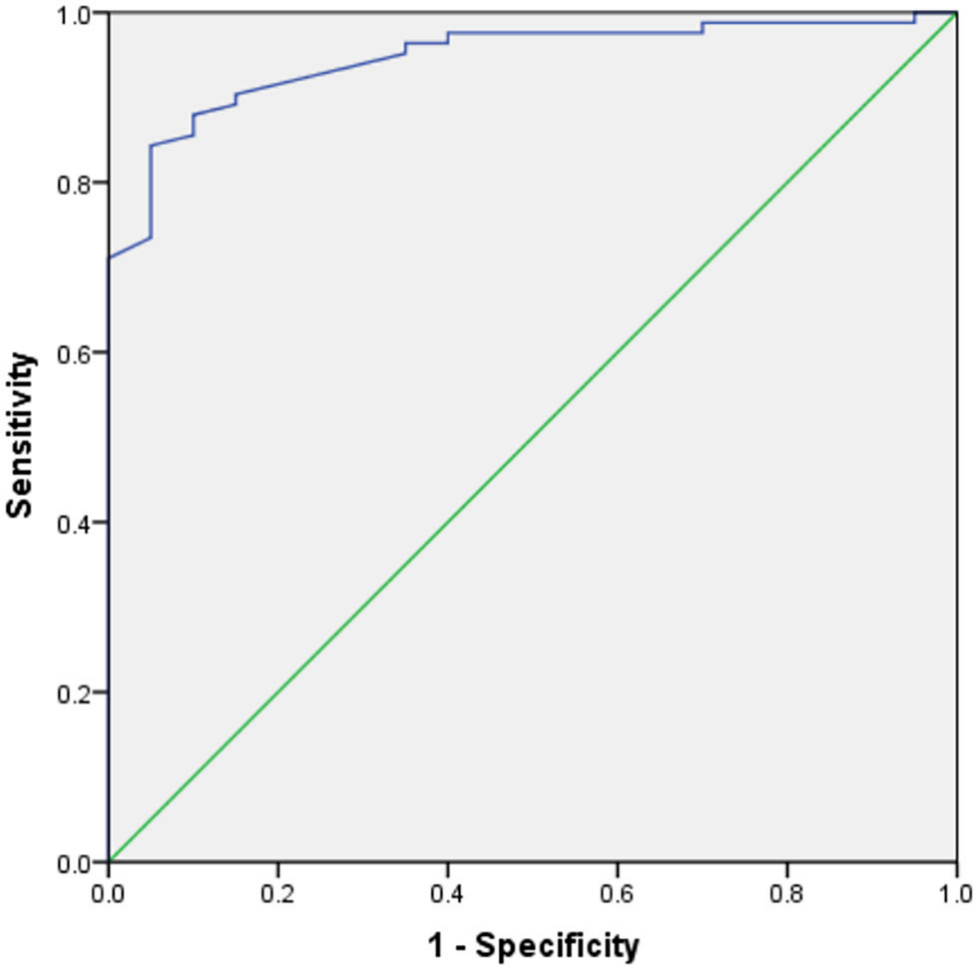
ROC curves for the prediction of DN using serum Zn concentration.

**Figure 2:**
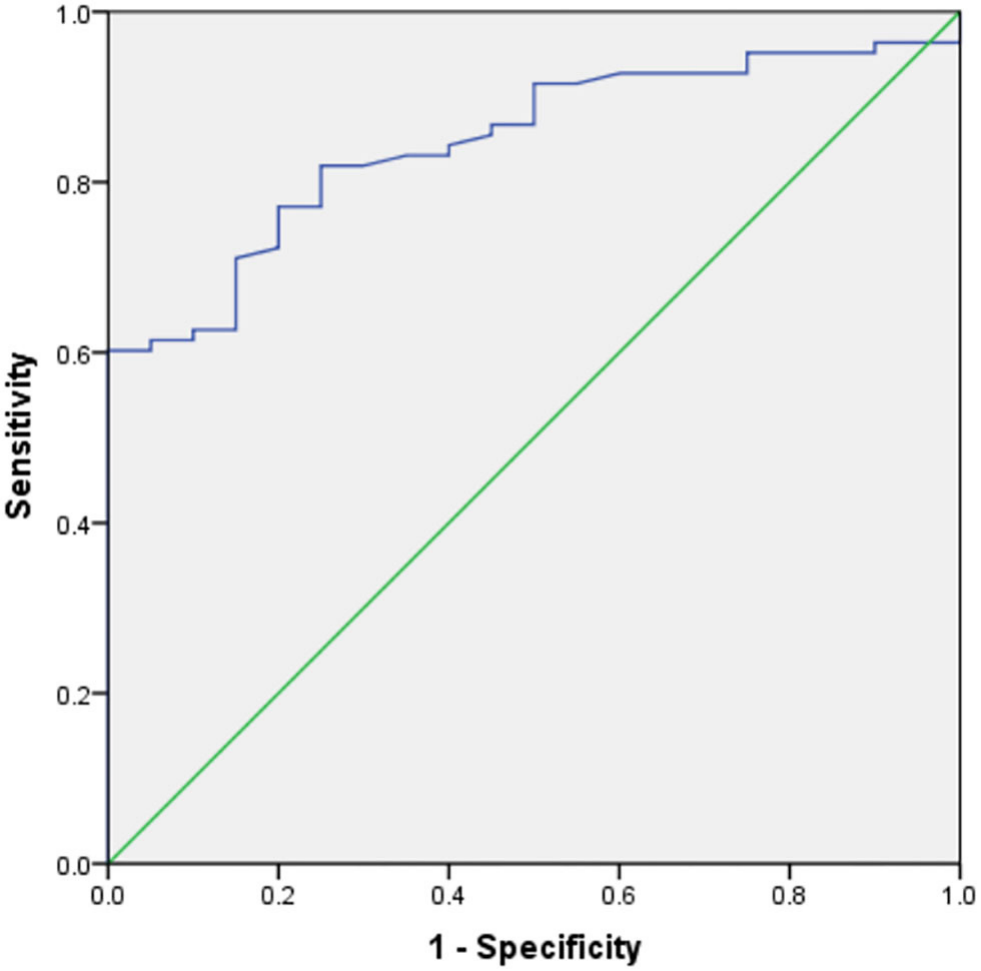
ROC curves for the prediction of DN using serum FPG concentration.

**Figure 3:**
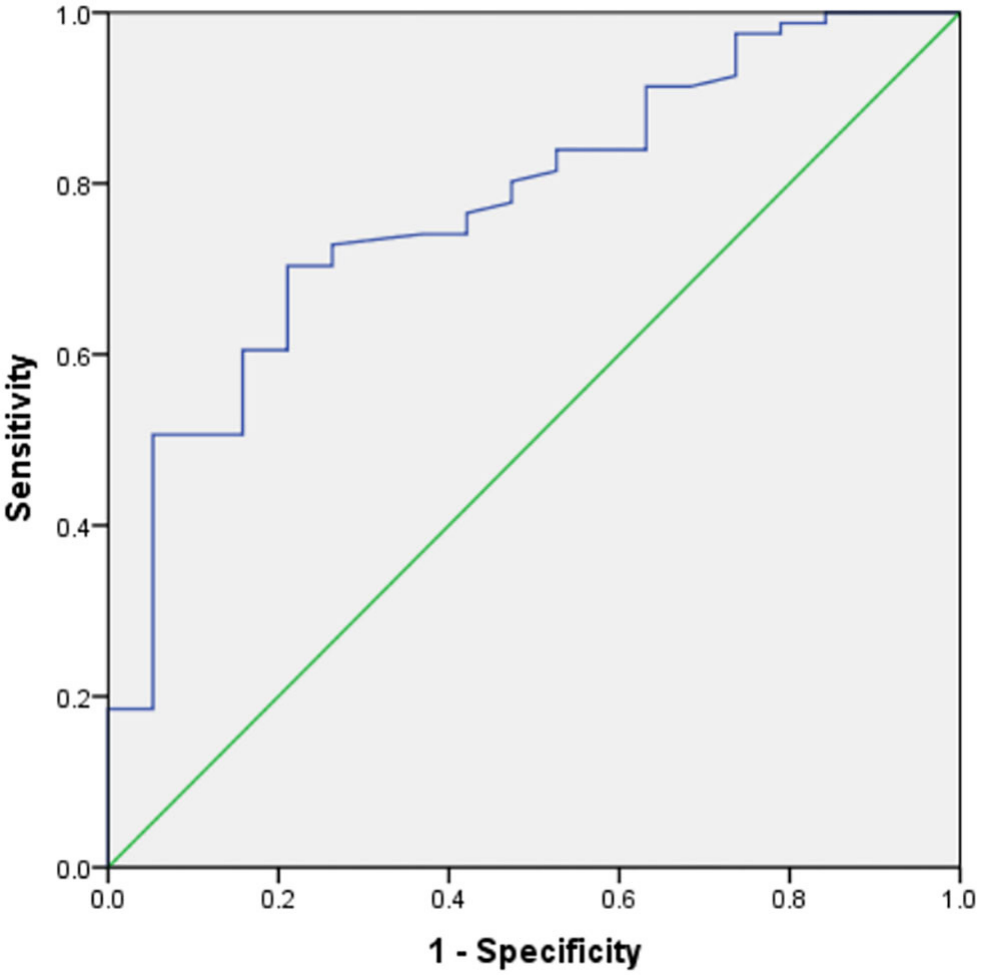
ROC curves for the prediction of DN using serum IL-10 concentration.

Table 3 shows the results of an evaluation of possible predictors of T2DN. The sensitivity and specificity for the prediction of T2DN using serum Zn concentration were 84.3 and 95%, respectively; those for the prediction of T2DN using FPG concentration were 82.9 and 78.9%; and those for the prediction of T2DN using IL-10 were 70.7 and 78.9%. Figures 1–3 show the receiver operating characteristic (ROC) curves for the prediction of T2DN using serum Zn, FPG, and IL-10, respectively.

**Table 3:** Sensitivity and specificity for potential predictors of DN by ROC curve analysis.

**Item**	**AUC**	**Sensitivity (%)**	**Specificity (%)**	***P***
Serum Zn (mmol/L)	0.946	84.3	95.0	<0.001
FPG (mmol/L)	0.841	82.9	78.9	<0.001
IL-10 (pg/mL)	0.780	70.7	78.9	<0.001

*P < 0.05 indicates a statistically significant difference. FPG, fasting plasma glucose; Zn, zinc; IL-10, interleukin-10*.

### Demographic and Biochemical Parameters of Patients With Different Degrees of DN

Table 4 shows that there were no significant differences in sex ratio; history of smoking; BMI; or the serum Mg, IL-1β, IL-12, TNF-α, or CRP concentrations between the T2DM and T2DN subgroups. The macroalbuminuria and renal failure groups had higher prevalences of a history of hypertension, and higher IL-6 and IL-8 concentrations, but lower serum Zn, 24 h urine Zn loss, 24 h urine Mg loss, and eGFR than the normal and microalbuminuria groups (*P* < 0.05). In addition, the microalbuminuria and renal failure groups had lower IL-10 concentrations than the normal group (*P* < 0.05), and the microalbuminuria group also had a lower IL-10 concentration than the macroalbuminuria group (*P* < 0.05).

**Table 4:** Demographic and biochemical parameters of patients with different degrees of DN.

**Item**	**Normal group** ** (*n* = 24)**	**Microalbuminuria group** ** (*n* = 18)**	**Macroalbuminuria group** ** (*n* = 24)**	**Renal failure group** ** (*n* = 17)**	***P***
Age (years)	52.67 ± 10.07	57.89 ± 7.45	56.79 ± 8.56	62.35 ± 11.11^*^	0.017
Male, *n* (%)	17 (70.8)	14 (77.8)	14 (58.3)	11 (64.7)	0.579
BMI (kg/m^2^)	25.51 (24.18–27.25)	26.87 (25.05–28.51)	27.04 (24.60–28.37)	25.78 (23.24–28.20)	0.302
Hypertension, *n* (%)	11 (45.8)	13 (72.2)	22 (91.7)^*^^∞^	16 (94.1)^*^^∞^	0.001
Smoking, *n* (%)	9 (39.1)	7 (38.9)	11 (45.8)	4 (23.5)	0.543
Serum Zn (mmol/L)	10.85 (8.95–11.95)	10.40 (8.63–11.50)	7.50 (5.48–8.85)^*^^∞^	8.10 (5.95–8.65)^*^^∞^	<0.001
24 h urine Zn (mmol/L)	10.68 ± 4.10	10.34 ± 4.48	8.19 ± 4.69^*^	3.68 ± 3.17^*^^∞^^&^	<0.001
Serum Mg (mmol/L)	0.89 ± 0.08	0.84 ± 0.10	0.84 ± 0.09	0.88 ± 0.10	0.243
24 h urine Mg (mmol/L)	3.65 ± 1.40	3.70 ± 1.49	2.80 ± 1.11^*^^∞^	2.62 ± 1.31^*^^∞^	0.018
IL-1β (pg/mL)	2.07 (1.30–2.88)	1.07 (0.51–2.51)	1.43 (0.95–2.61)	0.92 (0.44–1.49)	0.053
IL-6 (pg/mL)	3.43 (2.49–4.03)	2.88 (2.23–5.04)	5.57 (3.65–7.32)^*^^∞^	7.65 (6.47–10.38)^*^^∞^^&^	<0.001
IL-8 (pg/mL)	5.09 ± 1.41	5.29 ± 2.22	7.61 ± 2.19^*^^∞^	8.99 ± 2.05^*^^∞^^&^	<0.001
IL-10 (pg/mL)	2.11 ± 0.79	1.29 ± 0.48^*^	1.77 ± 0.73^∞^	1.58 ± 0.80^*^	<0.001
IL-12 (pg/mL)	0.08 (0.00–0.83)	0.13 (0.00–0.62)	0.37 (0.02–0.92)	0.31 (0.00–0.46)	0.425
TNF-α (pg/mL)	1.75 ± 1.15	2.09 ± 2.53	1.80 ± 0.85	1.24 ± 0.79	0.157
CRP (mg/L)	3.23 (3.13–3.23)	3.23 (3.21–3.40)	3.23 (3.13–3.72)	5.38 (3.23–7.24)	0.225
eGFR (ml min^−1^.1.73 m^−2^)	112.83 ± 39.54	94.15 ± 31.39^*^	44.19 ± 21.70^*^^∞^^&^	10.48 ± 2.72^*^^∞^^&^	<0.001

* *vs. the normal group P < 0.05*;

∞ *vs. the microalbuminuria group P < 0.05*;

& *vs. the macroalbuminuria group P < 0.05. P < 0.05 means a statistically significant difference. BMI, body mass index; Zn, zinc; Mg, magnesium; IL, interleukin; CRP, C-reactive protein; TNF-α, tumor necrosis factor-α; eGFR, estimated glomerular filtration rate*.

### Correlation Coefficients Between Zn and Mg Concentrations and Indices of Clinical Parameters and Renal Function in Patients With T2DN

Table 5 lists the correlations between Zn concentration and indices of clinical parameters and renal function in patients with T2DN. There were positive correlations between serum Zn and 24 h urine Zn, history of hypertension, and eGFR *(P* < 0.05), and negative correlations between a history of smoking, SCr, and ACR and 24 h urine albumin loss (*P* < 0.05).

**Table 5:** Correlation coefficients between Zn and clinical parameters and indices of renal function in DN patients.

**Item**	**Serum Zn**		**24 h urine Zn**	
	***r***	***P***	***r***	***P***
Age (years)	−0.185	0.094	−0.061	0.503
BMI (kg/m^2^)	0.004	0.972	0.077	0.492
Hypertension, *n* (%)	0.837	<0.001	−0.058	0.602
Smoking, *n* (%)	−0.272	0.013	0.318	0.003
Serum Zn (mmol/L)	1		0.423	<0.001
24 h urine Zn (mmol/L)	0.423	<0.001	1	
SCr (μmol/L)	−0.522	<0.001	−0.467	<0.001
24 h urine albumin (mg/24 h)	−0.573	<0.001	−0.342	<0.001
ACR (mg/g)	−0.554	<0.001	−0.430	<0.001
eGFR (ml min^−1^ 1.73 m^−2^)	0.557	<0.001	0.518	<0.001

*P < 0.05 means a statistically significant difference. BMI, body mass index; Zn, zinc; SCr, serum creatinine; ACR, albumin/creatinine ratio; eGFR, estimated glomerular filtration rate*.

Table 6 shows the correlations between serum Mg concentration and indices of clinical parameters and renal function in patients with T2DN. There were positive correlations between serum Mg and a history of hypertension and smoking. Serum Mg concentration was not related to renal function in patients with T2DN. However, there were positive correlations between 24 h urine Mg concentration and eGFR and a history of hypertension (*P* < 0.05) and a negative correlation between 24 h urine Mg concentration and ACR and a history of smoking (*P* < 0.05).

**Table 6:** Correlation coefficients between Zn and clinical characteristics and indices of renal function in DN patients.

**Item**	**Serum Mg**		**24 h urine Mg**	
	***r***	***P***	***r***	***P***
Age (years)	0.172	0.120	−0.103	0.352
BMI (kg/m^2^)	−0.132	0.237	−0.072	0.523
Hypertension, *n* (%)	−0.315	0.004	0.336	0.002
Smoking, *n* (%)	−0.849	<0.001	−0.994	<0.001
Serum Mg (mmol/L)	1		0.201	0.068
24 h urine Mg (mmol/L)	0.201	0.068	1	
SCr (μmol/L)	0.008	0.430	−0.206	0.063
24 h urine albumin (mg/24 h)	−0.171	0.121	−0.197	0.175
ACR (mg/g)	−0.115	0.303	−0.431	0.002
eGFR (ml min^−1^ 1.73 m^−2^)	−0.041	0.712	0.268	0.014

*P < 0.05 indicates a statistically significant difference. Mg, magnesium; SCr, serum creatinine; ACR, albumin/creatinine ratio; eGFR, estimated glomerular filtration rate*.

### Analysis of Correlations Between Zn and Mg Concentrations and Serum Pro-inflammatory Cytokine Concentrations in Patients With T2DN

Table 7 shows that there were negative correlations of serum Zn concentration with IL-6 and IL-8 (*P* < 0.05), and of 24 h urine Zn loss with IL-6 and IL-8 (*P* < 0.05). Table 8 shows that there were no correlations between Mg concentration and serum pro-inflammatory cytokine concentrations in patients with T2DN.

**Table 7:** Correlation coefficients between Zn and serum pro-inflammatory cytokines in DN patients.

**Cytokine**	**Serum Zn**		**24 h urine Zn**	
	***r***	***P***	***r***	***P***
IL-1β (pg/mL)	0.067	0.646	0.167	0.132
IL-6 (pg/mL)	−0.466	<0.001	−0.261	0.017
IL-8 (pg/mL)	−0.514	<0.001	−0.320	0.004
IL-10 (pg/mL)	−0.030	0.788	0.058	0.607
IL-12 (pg/mL)	−0.134	0.232	−0.067	0.551
TNF-α (pg/mL)	−0.086	0.454	0.139	0.225
CRP (mg/L)	−0.190	0.085	−0.081	0.465

*P < 0.05 indicates a statistically significant difference. Zn, zinc; IL, interleukin; TNF-α, tumor necrosis factor-α; CRP, C-reactive protein*.

**Table 8:** Correlation coefficients between Mg and serum pro-inflammatory cytokines in DN patients.

**Cytokine**	**Serum Mg**		**24 h urine Mg**	
	***r***	***P***	***r***	***P***
IL-1β (pg/mL)	0.051	0.647	0.122	0.272
IL-6 (pg/mL)	0.001	0.991	−0.182	0.099
IL-8 (pg/mL)	−0.020	0.859	−0.162	0.147
IL-10 (pg/mL)	0.126	0.262	0.135	0.230
IL-12 (pg/mL)	−0.002	0.984	0.084	0.455
TNF-α (pg/mL)	0.005	0.968	0.093	0.416
CRP (mg/L)	−0.064	0.567	−0.081	0.465

*P < 0.05 indicates a statistically significant difference. Mg, magnesium; IL, interleukin; TNF-α, tumor necrosis factor-α; CRP, C-reactive protein*.

## Discussion

Although trace elements are found only in small quantities in the human body, they are extremely important for human health. A previous study of the relationship between DM and trace elements showed that appropriate homeostasis of trace elements assists with the control of blood glucose and slows tissue damage (18). The trace elements Zn and Mg are known to influence the development of diabetes and its complications. Zn is involved in the production, storage, and secretion of insulin by islet β-cells, and protects vascular endothelial integrity via its anti-oxidative, anti-apoptotic, and membrane stabilizing effects (19). In addition, Mg plays an important role in glucose homeostasis and the maintenance of insulin bioactivity (20).

Zn homeostasis is associated with DN, and in the present study we found that the serum Zn concentration of patients with DN is significantly lower than that of healthy individuals. Logistic regression and ROC analysis were used to identify predictors of DN, and both methods showed that serum Zn concentration is an independent and specific predictor of DN, which is a similar finding to that of Luo et al. (12).

We also found that in patients with macroalbuminuria or renal failure, the serum Zn concentration was significantly lower than in patients with T2DM but not DN, and in those with microalbuminuria. Furthermore, the serum Zn concentration in patients with DN negatively correlated with 24 h urinary albumin loss (*r* = −0.573); and the serum concentrations of IL-6 (*r* = −0.446) and IL-8 (*r* = −0.514). This suggests that Zn homeostasis is associated not only with DN-associated renal functional damage, but also with inflammatory responses in patients with DN. Similarly, in previous studies, Al-timimi et al. (21) found that serum Zn concentration decreases in patients with DN and that this is accompanied by a gradual decrease in eGFR and an increase in albuminuria. Furthermore, Zhang et al. (22) found that dietary Zn restriction significantly reduced the Zn concentrations in the plasma and kidneys of mice, and exacerbated tubulointerstitial fibrosis in diabetic mice. Khan et al. (23) demonstrated that dietary Zn supplementation reduces urinary albumin loss in patients with T2DM and DN, and Tang et al. (24) showed that dietary Zn supplementation prevents diabetes-related renal fibrosis and reduces 24 h urinary protein loss.

Previous studies have also shown that Zn administration reduces NF-κB activation and ameliorates vascular endothelial cell dysfunction (25). NF-κB is an important pro-inflammatory mediator in patients with DN, with its activation resulting in the production of a variety of pro-inflammatory cytokines, the proliferation, and hypertrophy of mesangial cells, and tubulointerstitial fibrosis (26). Specifically, NF-κB activation results in greater release of TNF-α, IL-6, and IL-8, and in turn, TNF-α increases the production of IL-1, IL-6, IL-8, and IL-18 (27). Three mechanisms have been suggested for the downregulation of NF-κB by Zn. Firstly, Zn may act as an immunomodulator to inhibit nucleotide phosphodiesterase, leading to an increase in cyclic-guanosine monophosphate concentration and the activation of protein kinase A, which prevents NF-κB entry into the nucleus and inhibits the phosphorylation of IκB kinase (IKK), and thereby NF-κB target gene transcription (28, 29). Secondly, zinc-regulated transporters, iron-regulated transporter-like protein-8 (ZIP8) may be involved. These are NF-κB targets that are significantly upregulated/ in inflammation. ZIP8 increases the intracellular concentration of Zn by importing it from the extracellular fluid and promoting its release from intracellular depots. Zn directly inhibits IKK via the thiol reaction. Thus, Zn can negatively regulate NF-κB pathways (30–32). Thirdly, serum Zn affects the expression of the Zn-finger protein A20. In the TNF receptor and toll-like receptor signaling pathways, A20 prevents IKK phosphorylation, inhibits NF-κB activation, and also acts on key upstream molecules that inhibit inflammatory signaling (11, 33). The regulation of ubiquitin-dependent signaling by A20 is dependent on serum Zn concentration. Prasad et al. (34) has shown that in monocytes, endothelial cells, and cancer cells, A20 expression is affected by serum Zn concentration, and Li et al. (35) found that Zn stimulates A20 transcription by causing epigenetic modification of the A20 promoter. Finally, Zn acts as a free radical scavenger, thereby contributing to the stability of A20 (11, 36). Thus, in summary, Zn homeostasis is closely related to the inflammatory response and the progression of DN, but the specific mechanisms involved require further study.

In the present study, we found that the urine Zn concentration of patients with DN is higher than that of healthy individuals, which may at least in part explain the low serum Zn concentration in patients with DN. This high urine zinc concentration may be related to polyuria, the blood glucose concentration, glycosuria, and/or proteinuria. With the progression of DN, the urine Zn concentrations gradually decreased, and when patients with DN had an eGFR <15 ml min^−1^ 1.73 m^−2^, this decrease became significant. This may be explained by a decrease in urine volume or even anuria in patients with renal failure, and abnormal Zn metabolism is more common in patients at this stage of progression of DN.

DN is often associated with Mg deficiency. In the present study, we also found that the serum Mg concentration of patients with T2DM and DN was significantly lower than that in healthy individuals. A recent study found that hypomagnesemia is associated with a high incidence of DN and is an independent predictor of DN (16), and others have shown that hypomagnesemia is associated with both the development and progression of DN (37, 38).

Mg is an important cofactor and activator of many enzymes that are involved in glucose metabolism. In the absence of Mg, these enzymes cannot utilize high-energy phosphate bonds, which prevents phosphorylation, thereby directly affecting carbohydrate metabolism (39). Mg deficiency adversely affects cell homeostasis and may cause endothelial dysfunction and thrombosis by increasing platelet aggregation and vascular calcification (40). It can also lead to the excessive production of free radicals, which can damage mitochondria and change their permeability, which accelerates cellular necrosis and apoptosis (41), and can reduce the activity of antioxidant enzymes (42, 43), all of which are factors that facilitate the development of DN.

In the present study, there was no difference in the urine Mg concentration between patients with DN and healthy controls, which is not consistent with the results of previous studies (44). When healthy people eat a normal diet, about 30–50% of Mg can be absorbed, and the higher their energy intake, the larger the amount of Mg that is absorbed. Furthermore, dietary protein and amino acids also increase Mg absorption (45). Thus, long-term dietary therapy in DN may reduce Mg intake. Furthermore, gastroparesis and diarrhea caused by diabetic autonomic neuropathy can also reduce Mg absorption in the gastrointestinal tract (46). Normally, the kidneys are important regulators of Mg homeostasis, and the renal excretion of Mg largely depends on the serum Mg concentration (47). We found that serum Mg concentration was lower in patients with DN. To maintain serum Mg homeostasis, the kidney reduces urinary Mg excretion. However, in the present study, we found no difference in urine Mg concentration between healthy controls and patients with DN. Therefore, urinary Mg excretion in patients with DN would appear to be affected by many factors and may not be an accurate guide to overall Mg status. Thus, patients with DN should be considered Mg deficient, even if their urine Mg concentrations are normal. With the progression of DN, urinary Mg excretion decreased gradually in the present study. This may be because in the early stages of DN, the eGFR is normal; there may even be hyperfiltration, but with the progression of DN the compensation for renal dysfunction becomes insufficient, and the urine Mg concentration decreases with the volume of urine excreted, such that ultimately urine Mg is significantly reduced. Future studies will be directed toward collecting more information on the daily dietary habits of patients with DN and exploring the metabolism and distribution of trace elements Mg and Zn in DN patients, such as their cellular concentrations, as well as the concentrations of Mg and Zn in their diets. This will contribute to the development of appropriate evaluation indicators of the precise levels of trace elements in DN patients.

In summary, in the present study, we have demonstrated that the serum concentrations of the trace elements Zn and Mg in patients with T2DM and DN are significantly lower than those of healthy individuals. Serum Zn was found to be an independent predictor of DN and to have a protective role. Furthermore, the serum Zn, urine Zn, and urine Mg concentrations were found to decrease with the progression of DN-associated renal functional damage. Hypozincemia may be associated with DN-associated renal functional damage because it exacerbates renal inflammation. This study has several limitations. First, because it was a cross-sectional study, it was not possible to determine whether hypozincemia was a pathogenic factor or solely a risk biomarker. Second, due to time constraints, the sample size in our study was too small. In future studies, we will increase the sample size to improve the accuracy of the results.

## Data Availability Statement

The original contributions presented in the study are included in the article/supplementary material, further inquiries can be directed to the corresponding author/s.

## Ethics Statement

The studies involving human participants were reviewed and approved by Ethics committee of the First Hospital of Jilin University. The patients/participants provided their written informed consent to participate in this study.

## Author Contributions

WS, JF, and YJ designed the experiments. WS and JF wrote the paper. JF, HW, ZJ, YW, WW, and WS performed the experiments and collected and analyzed the data. All authors read and approved the final manuscript.

## Conflict of Interest

The authors declare that the research was conducted in the absence of any commercial or financial relationships that could be construed as a potential conflict of interest.

## Abbreviations

Zn: Zinc
Mg: magnesium
OS: oxidative stress
DN: diabetic nephropathy
T2DM: type 2 diabetes mellitus
IL: interleukin
TNF-α: tumor necrosis factor-α
NF-κB: nuclear factor kappa-B
ACR: albumin/creatinine ratio
FPG: fasting plasma glucose
eGFR: estimated glomerular filtration rate
BUN: blood urea nitrogen
Scr: serum creatinine
TC: total cholesterol
TG: triglyceride
IKK: IκB kinase
ZIP8, zinc-regulated transporters: iron-regulated transporter-like protein-8.
